# The attitudes and feelings of mental health nurses towards adolescents and young adults with nonsuicidal self-injuring behaviors

**DOI:** 10.1186/s13034-020-00343-5

**Published:** 2020-09-22

**Authors:** Matejka Pintar Babič, Branko Bregar, Maja Drobnič Radobuljac

**Affiliations:** 1 Center for Mental Health, University Psychiatric Hospital Ljubljana, Grablovičeva 44a, 1000, Ljubljana, Slovenia; 2 Angela Boškin Faculty of Health Care, Jesenice, Slovenia; 3 Medical Faculty, University of Ljubljana, Ljubljana, Slovenia

**Keywords:** Cutting, Self-mutilation, Emotions, Nurse, Adolescents, Young adults, Self-harm, Non-suicidal self-injury, Emergency psychiatry, Psychotherapeutic unit

## Abstract

**Background:**

Attitudes towards patients with self-harm behaviors are decisive for the quality of the relationship of healthcare professionals towards them, which is further linked to successful treatment. In mental health settings, nurses are the ones spending the longest time caring for these patients. Nurses often experience negative emotions while delivering care which may lead to professional burnout and suboptimal patient care. The purpose of this study was to explore the feelings and attitudes of nurses working in different psychiatric hospital settings toward adolescents and young adults with non-suicidal self-injury (NSSI).

**Subjects and methods:**

The subjects were nurses from the tertiary psychiatric hospital who deliver mental health care to patients with NSSI on a daily basis (n = 76; 20 males, 56 females; average age 42 ± 8 years; average working experience 20 ± 9 years). Data were collected via a self-report questionnaire consisting of three parts (sociodemographic data, Emotional Burden, Adapted Self-Harm Antipathy-Scale). In the latter two parts of the questionnaire, the subjects rated their level of agreement with the emotions and statements on a five-point Likert scale. Nonparametric tests were used for statistical analysis. The statistical significance was set to *p* < *0.05*.

**Results:**

The emotions of nurses towards patients with NSSI were not very negative and the attitudes were positive. Powerlessness was the most prevalent (3.55 ± 1.038) of the studied emotions, followed by uncertainty (3.21 ± 1.225). The subjects disagreed with feeling anger (2.34 ± 1.17) and despair (2.07 ± 1.09) and were undecided about being afraid (3.07 ± 1.2). The nurses with higher education felt more negative emotions than those with medium education. Education did not affect nurses’ attitudes. The nurses from non-psychotherapeutic units felt more negative emotions and less positive attitudes than those from psychotherapeutic units. Gender did not affect the emotions felt towards patients, but the female nurses held more positive and less negative attitudes.

**Conclusions:**

The respondents expressed low levels of negative emotions and positive and caring attitudes towards patients with NSSI, indicating a good predisposition for empathetic work and long-term burnout prevention. However, the differences observed with regards to education, gender and especially working environment indicate the different needs for environmental, educational and supervisory support.

## Background

People deliberately self-harm for various reasons, some of which are entwined with cultural or environmental standards or rituals, while others are pathological [1]. The National Institute for Health and Care Excellence (NICE) guideline defines deliberate self-harm (DSH) as “self-poisoning or self-injury, irrespective of the apparent purpose of the act. An individual episode of self-harm might be an attempt to end life. However, many acts of self- harm are not directly connected to suicidal intent” [2]. A subgroup of this behavior, non-suicidal self-injury (NSSI), is defined as “a deliberate causing of damage to one’s own body in a manner that is not socially acceptable and does not have a suicidal intent” [1, 3, 4]. As such, it occurs most frequently in the adolescent and young adult population and most commonly comprises cutting or slashing, although burning, self-battery, scratching, biting, wound interference and head banging are also common [5]. The majority of patients use this behavior with the aim of calming down, relaxing and gaining control over their emotional difficulties, with an intent opposite to an intention to die and perpetuated most commonly with negative reinforcement [1].

The lifetime prevalence of NSSI in adults is reported to be 6% (from 2 to 17%), with higher lifetime prevalence rates in younger age groups: 23% in young adults (aged 18–24) and 19% in adolescents (aged 10–17) [5, 6]. The lifetime prevalence of NSSI among the general population of Slovene high-school students (aged 15–19) is reported to be up to 24% [7]. In clinical samples the prevalence of DSH and NSSI is expectedly higher. Studies in clinical adolescent samples report the 12-month prevalence of NSSI to be around 50% [8, 9].

The attitude of health care workers towards patients, including stigmatization of patients, influences their daily work routine and the quality of their health care. This has been studied in various fields of health care and psychiatry [10, 11]. Negative attitudes, which are reported to significantly influence outcomes and are even connected to adverse events such as patient falls and medication errors, are especially problematic [12, 13]. The relationship between exposure to NSSI and attitudes towards young people who self-injure has not been widely explored. A review article of the attitudes of accident and emergency (A&E) staff towards adolescents who self-harm (attempted suicide and NSSI) reported that work setting, patient characteristics, and education and training all appeared to have an influence, although the results of the studies were inconsistent and they concluded that more research is needed [14]. Another review examining nurses attitudes towards self-harm (without suicide) reported inconclusive results with regard to the influence of work experience, gender and age, however they found more positive attitudes in nurses working within mental health settings and among nurses with higher qualifications [15]. Cleaver and coworkers also reported that the fact that patients are younger positively influenced the attitudes of A&E and ambulance staff towards adolescents who self-harmed [16]. Studies on adolescents who self-harmed showed that school staff and nursing students’ attitudes towards these students were more positive with more experience [17, 18] although for the nurses working within forensic adolescent facilities and A&E staff without previous training in mental health, the duration of experience with patients with DSH negatively impacted their attitudes [19, 20]. More previous education and specific educational interventions were shown to improve the attitudes of professionals working with clients who self-injured [18, 20–23].

Importantly, Hasking et al. reported that fewer than 59% of adolescents confided their NSSI to someone else. Confidantes included friends (68.83%), parents (26.62%), mental health workers (13.64%), boy/girlfriends (11.69%), general practitioners (7.79%), siblings/cousins (3.25%), and teachers (3.25%). Over 40% of young people reporting non-disclosure of their NSSI to anyone highlights the intimate nature of NSSI and the need to encourage disclosure of this behavior [24].

The attitudes of nurses towards patients with these behaviors are especially important if patients are to confide and receive optimal care and treatment [15, 25]. Nurses caring for patients with NSSI work in a high stress environment. While they are expected to remain professional and offer support to the patients and their families regardless of the emotional weight of the situation, they face elevated risk for compassion fatigue and burnout, which negatively impacts their ability to empathize and, consequently, the patients’ experience with care [25, 26]. A lack of knowledge and understanding by healthcare workers about the etiology and aims of self-injury contributes to a negative perception of the individuals who self-injure and, due to this, a poor healthcare experience [17, 19, 22, 27–29]. Adults who self-harm describe their experience with professionals as judgmental, not listening and lacking sufficient knowledge, they report that a positive relationship with healthcare personnel is key to motivating patients with NSSI to continue treatment and seek further help [28]. Even though there is still a lack of available data on the experience of adolescents with NSSI, they report uncertainty in deciding to disclose [28].

Due to the fact that NSSI is so common in the adolescent and young adult population, the main priority is therefore to improve the relationship of healthcare personnel to these patients and, in order to improve it, the personnel most at risk for antipathy towards such patients should be identified and included in preventive interventions [25, 28]. To our knowledge, no other study has compared the experiences of nurses working in different psychiatric settings, the workplaces with the highest prevalence of NSSI.

### Purpose and goals

The goal of this study was to determine the emotions and attitudes of nurses working in different psychiatric settings towards adolescents and young adults with NSSI. We aimed to answer the following questions: (1) How frequently do psychiatric nurses have to deal with their own negative emotions while working with young patients with NSSI? (2) What are the attitudes of psychiatric nurses towards young patients with NSSI? (3) How are the emotions and attitudes of psychiatric nurses related to gender, workplace, education and religious status?

## Methods

### Procedure

A descriptive cross-sectional quantitative study took place in April and May 2015. Nurses at the University Psychiatric Hospital Ljubljana, the largest psychiatric in- and outpatient facility in Slovenia, filled out self-report questionnaires. Only nurses from the units with the highest frequencies of NSSI were approached and invited to participate in the study. They were divided into groups according to gender, education (high or medium education), religious status (active or not) and work setting (psychotherapeutic and non-psychotherapeutic settings). The group working in psychotherapeutic settings were the nurses from adolescent psychiatry and drug addiction open wards and the group working in non-psychotherapeutic setting were the nurses from outpatient clinics and female and male intensive secure units. The intensive units also admitted all the children and adolescents who needed secure settings, since there was no secure ward for children and adolescents in Slovenia until 2019 [30]. The adolescent unit, with 150 admissions per year, was the unit with the highest NSSI rate in the hospital. Some of the nurses only worked with adolescents (aged 14 – 22), others were asked to keep in mind only experiences with adolescents and adults up to 25 years old (students) during the evaluation. The Ethical Committee of the University Psychiatric Hospital Ljubljana approved the study in 2015. The cooperation was voluntary and anonymous. The subjects signed informed consents prior to enrollment.

### Subjects

Ninety nurses were invited to the study (31% of all nurses at the hospital) and given the questionnaires. Of these, 76 returned the questionnaires (84.4% participation rate): 20 were males (26%) and 56 females (74%). Their average age was 42 years (± 8 years), with an average of 20 years of working experience (± 9 years), the average duration of service at the present position was 10 years (± 7 years). Most of the subjects had higher education (39 subjects; 51%) followed by high school education (37; 49%). Thirty-three of the respondents (43%) worked exclusively with adolescents, while the remainder (43; 57%) worked with adolescents and/or young adults. Twenty-two subjects (29%) responded that they were religious and active, 27 (35.5%) religious but not actively so, and 27 (35.5%) were not religious.

### Description of the questionnaire used

The questionnaire used was comprised of three parts and designed on the basis of the reviewed literature in this field [3, 15, 21, 25]. The first part of the questionnaire collected socio-demographic and general data (gender, age, education, workplace, religion, duration of service, number of patients with NSSI treated). The second part, the Emotional Burden Questionnaire (EBQ), designed for the purpose of the present study, assessed the burden on the subjects based on five emotions defined on the basis of the most commonly studied emotions [31–33]. The participants answered the following questions: “Working with a patient with NSSI I feel afraid.”; “… uncertain.; “… powerless.”; “… angry.”; “… desperate.”. The third part was a translated and back-translated Self-Harm Antipathy Scale (SHAS) comprising 29 attitudinal items previously used to evaluate the attitudes of healthcare workers towards patients with a DSH diagnosis [21, 34]. In the second and third parts (EBQ and SHAS), the subjects marked their responses on a 5-point Likert scale (1 = I don't agree at all; 2 = I don't agree; 3 = I neither agree nor disagree; 4 = I agree; 5 = I completely agree). The questionnaire was firstly tested on ten nurses for length and understandability. The questions were corrected according to their remarks.

### Validity and reliability of the questionnaire

The EBQ comprised of five items about nurses’ feelings while working with patients with NSSI. The reliability of this section was modest *(α* = 0.772*)*.

For the SHAS, the original authors used 23 (out of a possible 29) attitudinal items, which they combined into 6 factors: 1) Competence Appraisal, 2) Care Futility, 3) Client Intent Manipulation, 4) Acceptance and Understanding, 5) Rights and Responsibilities and 6) Needs Function. The internal consistency of the original scale was *α* = 0.89, although the individual sections ranged from 0.52–0.81 [34]. The reliability of the entire scale for the present study was lower *(α* = 0.58*).* We improved the reliability of our scale (to *α* = 0.61*)* by excluding three statements: Question Nr. 30: “I am highly supportive towards clients who self-harm “, Question Nr. 8: “An individual has the right to self-harm” and Question Nr. 17: “For some individuals self-harm can be a way of relieving tension”, a method allowed by the statistical program (SPSS version 20.0, SPSS Inc., Chicago, IL, USA). Hereinafter these three statements were no longer used in the analyses. The acquired data were not suitable for factor analysis according to the required conditions due to the small sample size [35], therefore we analyzed and interpreted each statement separately and didn’t use reverse scoring or calculate the cumulative scale.

### Data analysis

The Kolmogorov–Smirnov test performed for checking the normal distribution of data prior to the start of the statistical analysis was below 0.05 for all the observed variables, therefore nonparametric tests (the Mann–Whitney U-test, the Wilcox test, the Spearman test for correlation) were used for statistical analysis. The statistical significance was set to *p* < *0.05*.

## Results

All the subjects had interacted with patients with NSSI (n = 76, 100%) as a part of their work. Of these, 17 (22%) had encountered patients with acute NSSI (self-harming during their shifts) 1—4 times, 16 (21%) had had 5—8 encounters with patients with acute NSSI and 43 (57%) had had 9 or more encounters with such patients. The nurses working in psychotherapy settings had more experience caring for patients with NSSI (72.7%, 24/33 nurses reported 9 or more encounters) than those from non-psychotherapeutic units (44.2%, 19/43 nurses) (χ2 = 9.623, p = 0.008, df = 2). The methods of NSSI they encountered were cutting the skin (97%), wound interference (82%), banging against hard objects (78%), causing burns (63%), pulling hair (35%), or other methods (30%).

### Nurses’ emotions

The respondents specified that while treating patients with NSSI they most commonly felt powerless and disagreed most with feeling despair. They were on average undecided about feeling afraid. The standard deviation was high for all emotions but highest for “Uncertainty” (Table 1).

**Table 1:** Nurses’ emotions during treatment of patients with NSSI

	Average	SD	Comparison to “Powerlessness”	
			*Z*	*p*
I feel powerless	3.55	1.038		
I face uncertainty	3.21	1.225	30.084	*0.002*
I feel fear	3.07	1.204	3.700	* < 0.001*
I feel anger	2.34	1.172	5.864	* < 0.001*
I feel despair	2.07	1.087	6.595	* < 0.001*

The emotions were determined using a 5-point Likert scale (5 = I completely agree; 1 = I don't agree at all); *SD* standard deviation; Z—the Wilcox pair nonparametric test was applied for the comparison of “powerlessness” with each of the other 4 remaining emotions. Statistically significant differences (*p* < 0.05) are in italic

There were no statistically significant differences according to gender and religious status in how the subjects felt when working with patients with NSSI. However, in a statistically significant way, subjects with higher education more commonly agreed with feeling powerless or uncertain and disagreed less with feeling despair as compared to those with a middle-level education (Table 2). In a statistically significant way, with regard to workplace, the subjects employed on non-psychotherapeutic units agreed more commonly that they felt uncertain or afraid and disagreed less with feeling despair as compared to those working in psychotherapeutic units (Table 2).

**Table 2:** Nurses’ emotions during treatment of patients with NSSI with regard to gender, education, workplace and religiousness

Emotion	Gender		Education		Workplace		Religiousness		Gender	Education	Workplace	Religiousness
	Female (n = 56)	Male (n = 20)	High (n = 39)	Middle (n = 37)	Psych (n = 33)	Non-P (n = 43)	$\mathrm{Active }$(n = 22)	Non-R (n = 54)	Statistical significance (*p*)			
I feel fear	3.18 ± 1.208	2.75 ± 1.164	3.13 ± 1.031	3.00 ± 1.374	*2.67 ± 1.164*	*3.37 ± 1.155*	3.04 ± 1.136	3.11 ± 1.340	0.162	0.809	*0.011*	0.757
I feel powerless	3.62 ± 1.105	3.35 ± 0.813	*3.90 ± 0.821*	*3.19 ± 1.126*	3.36 ± 1.141	3.70 ± 0.939	3.59 ± 1.039	3.48 ± 1.051	0.16	*0.005*	0.177	0.610
I feel anger	2.39 ± 1.171	2.20 ± 1.196	2.49 ± 1.189	2.19 ± 1.151	2.15 ± 1.093	2.49 ± 1.222	2.27 ± 1.132	2.48 ± 1.252	0.089	0.259	0.233	0.458
I feel despair	2.12 ± 1.113	1.90 ± 1.021	*2.51 ± 1.167*	*1.59 ± 0.762*	*1.73 ± 0.977*	*2.33 ± 1.107*	2.02 ± 1.031	2.15 ± 1.199	0.486	* < 0.001*	*0.011*	0.767
I face uncertainty	3.34 ± 1.180	2.85 ± 1.309	*3.51 ± 1.073*	*2.89 ± 1.308*	*2.82 ± 1.310*	*3.51 ± 1.077*	3.20 ± 1.207	3.22 ± 1.281	0.428	*0.045*	*0.015*	0.915

N = 76; The data are presented as average ± standard deviation and p values (Mann–Whitney U-test*); n* number, Education: High – technical, university level or post-graduate / Middle—high school; Workplace: Psych—psychotherapeutic unit / Non-P—non-psychotherapeutic unit; Religiousness: Active – actively religious / Non-R—not actively religious; respondents replied on a 5-point Likert scale (5 = I completely agree; 1 = I don't agree at all). Statistically significant differences (*p* < 0.05) are in italic

### Nurses attitudes

When evaluating the attitudes towards patients with NSSI, the subjects agreed most strongly with the statement “I listen fully to self-harming clients’ problems and experiences.”, followed by the statements “I demonstrate warmth and understanding to self-harming clients in my care.”, “I feel concern for the self-harming client.”, “I acknowledge self-harming clients’ qualities.“, and “I help self-harming clients feel positive about themselves.” (Table 3), namely all of the statements assessing positive attitudes (reverse scored in the original SHAS) [34]. The respondents disagreed most with the statements “A self-harming client is a complete waste of time.”, “I feel critical towards self-harming clients.”, “Self-harming clients have only themselves to blame for their situation.”, “I find it rewarding to care for self-harming clients.”, and “People who self-harm lack solid religious convictions.” (Table 3). Apart from the statement “I find it rewarding to care for self-harming clients.” all the most disagreed with statements assessed negative attitudes [34]. The largest standard deviation was found for the statements “People who self-harm are usually trying to get sympathy from others.” and “I feel to blame when my clients self-harm.”, although on average the participants disagreed with these two statements (Table 3), both assessing negative attitudes [34].

**Table 3:** The attitudes of the nurses towards patients with NSSI and differences according to gender, education, workplace and religious status

Statements regarding the attitude	x	s	Gender		Education		Workplace		Religiousness		G (p)	E (p)	W (p)	R (p)
			F (n = 38)	M (n = 14)	H (n = 27)	M (n = 25)	P (n = 28)	N –P (n = 24)	R (n = 22)	N –R (n = 54)				
20. I listen fully to self-harming clients’ problems and experiences*	4.64	0.74	4.71 ± 0.456	4.45 ±1.234	4.54 ± 0.942	4.67 ± 0.435	4.64 ± 0.994	4.65 ± 0.482	4.86 ± 0.351	4.56 ± 0.839	0.952	0.459	0.163	0.077
23. I demonstrate warmth and understanding to self-harming clients in my care*	4.61	0.49	4.64 ± 0.483	4.50 ± 0.513	4.62 ± 0.493	4.59 ± 0.498	4.70 ± 0.467	4.53 ± 0.505	4.68 ± 0.477	4.57 ± 0.499	0.265	0.854	0.155	0.384
26. I acknowledge self-harming clients’ qualities*	4.43	0.70	4.46 ± 0.687	4.35 ± 0.745	4.56 ± 0.598	4.30 ± 0.777	*4.67 ± 0.540*	*4.26 ± 0.759*	4.36 ± 0.848	4.46 ± 0.636	0.542	0.126	*0.011*	0.852
21. I feel concern for the self-harming client*	4.51	0.55	4.55 ± 0.537	4.40 ± 0.598	4.56 ± 0.502	4.64 ± 0.605	4.58 ± 0.502	4.47 ± 0.592	4.59 ± 0.503	4.48 ± 0.574	0.314	0.524	0.475	0.495
28. I can really help self-harming clients*	3.59	0.77	3.59 ± 0.804	3.60 ± 0.681	3.56 ± 0.788	3.62 ± 0.758	3.67 ± 0.595	3.53 ± 0.882	3.50 ± 0.859	3.63 ± 0.734	0.799	0.657	0.389	0.692
24. I help self-harming clients feel positive about themselves*	4.30	0.65	4.27 ± 0.700	4.40 ± 0.503	4.18 ± 0.756	4.43 ± 0.502	4.27 ± 0.574	4.33 ± 0,715	4.36 ± 0.727	4.28 ± 0.628	0.596	0.176	0.510	0.436
10. There is no way of reducing self-harm behaviors	1.82	0.81	1.80 ± 0.818	1.85 ± 0.813	1.85 ± 0.844	1.78 ± 0.787	*1.42 ± 0.792*	*2.12 ± 0.697*	1.77 ± 0.685	1.83 ± 0.863	0.755	0.823	*˂0.001*	0.956
7. A self-harming client is a complete waste of time	1.30	0.46	1.30 ± 0.464	1.30 ± 0,470	1.36 ± 0.486	1.24 ± 0.435	1.24 ± 0.435	1.35 ± 0.482	1.23 ± 0.429	1.33 ± 0.476	0.976	0.276	0.320	0.365
4. Self-harming clients do not respond to care	1.58	0.75	1.57 ± 0.806	1.60 ± 0.598	1.49 ± 0.683	1.68 ± 0.818	1.42 ± 0.663	1.70 ± 0.803	1.41 ± 0.666	1.65 ± 0.781	0.515	0.323	0.115	0.197
9. Self-harm is a serious moral wrongdoing	1.61	0.77	1.59 ± 0.757	1.65 ± 0.813	1.46 ± 0.643	1.76 ± 0.863	*1.30 ± 0.529*	*1.84 ± 0.843*	1.64 ± 1.002	1.59 ± 0.659	0.767	0.130	*0.002*	0.591
16. Self-harming clients have only themselves to blame for their situation	1.54	0.74	1.57 ± 0.783	1.45 ± 0.605	1.44 ± 0.598	1.65 ± 0.857	1.45 ± 0.617	1.60 ± 0.821	1.59 ± 0.959	1.52 ± 0.637	0.697	0.365	0.554	0.754
1. People who self-harm are usually trying to get sympathy from others	2.20	1.36	2.21 ± 1.552	2.15 ± 0.745	2.23 ± 1.667	2.16 ± 0.928	1.85 ± 0.667	2.47 ± 1.667	2.23 ± 0.973	2.19 ± 1.493	0.497	0.561	0.053	0.570
6. People who self-harm are typically trying to get even with someone	1.83	0.79	1.77 ± 0.786	2.00 ± 0.795	1.72 ± 0.793	1.95 ± 0.780	1.76 ± 0.792	1.88 ± 0.793	1.73 ± 0.767	1.87 ± 0.802	0.229	0.192	0.489	0.492
5. When individuals self-harm, it is often to manipulate carers	2.26	0.90	2.29 ± 0.929	2.20 ± 0.834	2.13 ± 0.801	2.41 ± 0.985	2.21 ± 0.740	2.30 ± 1.013	2.45 ± 0.963	2.19 ± 0.870	0.708	0.272	0.807	0.351
15. A self-harming client is a person who is only trying to get attention	2.21	0.91	2.20 ± 0.942	2.25 ± 0.851	2.15 ± 0.961	2.27 ± 0.871	2.24 ± 0.792	2.19 ± 1.006	2.41 ± 0.908	2.13 ± 0.912	0.642	0.485	0.660	0.204
18. Self-harming clients have a great need for acceptance and understanding*	4.20	0.83	*4.29 ± 0.847*	*3.95 ± 0.759*	4.13 ± 0.978	4.27 ± 0.652	4.27 ± 0.839	4.14 ± 0.833	4.36 ± 0.658	4.13 ± 0.891	*0.045*	0.909	0.402	0.386
11. People who self-harm lack solid religious convictions	1.57	0.84	1.48 ± 0.713	1.80 ± 1.105	1.54 ± 0.913	1.59 ± 0.762	1.45 ± 0.869	1.65 ± 0.813	1.68 ± 0.839	1.52 ± 0.841	0.313	0.475	0.181	0.348
2. People should be allowed to self-harm in a safe environment	1.66	0.93	1.71 ± 0.929	1.50 ± 0.946	1.85 ± 1.014	1.46 ± 0.803	1.52 ± 0.834	1.77 ± 0.996	1.59 ± 0.854	1.69 ± 0.968	0.199	0.075	0.228	0.763
12. Self-harm may be a form of reassurance for the individual that they are really alive and human*	2.83	1.06	*2.98 ± 1.036*	*2.40 ± 1.046*	3.03 ± 1.013	2.62 ± 1.089	2.94 ± 1.116	2.74 ± 1.026	2.68 ± 0.955	2.89 ± 1.093	*0.030*	0.108	0.266	0.347
14. Acts of self-harm are a form of communication to their situation*	3.93	0.75	*4.05 ± 0.749*	*3.60 ± 0.61*	4.00 ± 0.725	3.86 ± 0.787	4.06 ± 0.788	3.84 ± 0.721	3.77 ± 0.813	4.00 ± 0.727	*0.021*	0.411	0.170	0.245
13. Self-harming individuals can learn new ways of coping*	4.13	1.00	4.14 ± 1.017	4.10 ± 1.017	4.23 ± 1.012	4.03 ± 1.040	*4.42 ± 0.840*	*3.91 ± 1.109*	4.32 ± 0.945	4.06 ± 1.054	0.878	0.241	*0.022*	0.217
19. A self-harming client deserves the highest. standards of care on every occasion*	3.88	0.98	3.93 ± 1.006	3.75 ± 0.910	4.05 ± 0.826	3.70 ± 1.102	3.79 ± 1.023	3.95 ± 0.950	4.00 ± 1.069	3.83 ± 0.947	0.350	0.188	0.493	0.345
22. I feel critical towards self-harming clients	1.51	0.72	1.54 ± 0.738	1.45 ± 0.686	1.54 ± 0.756	1.49 ± 0.692	1.36 ± 0.653	1.63 ± 0.757	1.32 ± 0.568	1.59 ± 0.765	0.654	0.801	0.079	0.141
25. I feel to blame when my clients self-harm	2.57	1.21	*2.79 ± 1.155*	*1.95 ± 1.191*	2.67 ± 1.084	2.46 ± 1.346	*2.21 ± 1.083*	*2.84 ± 1.252*	2.82 ± 1.368	2.46 ± 1.145	*0.004*	0.304	*0.036*	0.311
27. I find it rewarding to care for self-harming clients*	1.55	0.75	1.59 ± 0.757	1.45 ± 0.759	1.59 ± 0.818	1.51 ± 0.692	1.67 ± 0.816	1.47 ± 0.702	1.73 ± 0.935	1.48 ± 0.666	0.394	0.858	0.280	0.408
29. I would feel ashamed if a member of my family engaged in self-harm	1.75	0.99	1.75 ± 1.031	1.75 ± 0.910	1.74 ± 0.938	1.76 ± 1.065	1.67 ± 0.890	1.81 ± 1.075	2.82 ± 0.935	1.72 ± 0.920	0.816	0.932	0.649	0.915

N = 76; The data are presented as average ± standard deviation and p—Mann–Whitney U-test statistical significance; Gender: M – male/F—female; Education: H – technical, university level or post-graduate / M—high school; Workplace: P—psychotherapeutic unit / N-P—non-psychotherapeutic unit; Religiousness: R – actively religious / N-R—not actively religious; respondents replied on a 5-point Likert scale (5 = I completely agree; 1 = I don't agree at all). *—questions measuring low antipathy (positive attitudes) in the original SHAS, the rest measure high antipathy (negative attitudes). Statistically significant differences (*p *< 0.05) are in italic. Questions 8, 17 and 30 were removed from the analyses.

There were no statistically significant differences with regards to the subjects’ *education or religious status*.

With regard to the *effect of gender on attitudes* towards patients with NSSI, female nurses agreed statistically significantly more than males with the statements: “Self-harming clients have a great need for acceptance and understanding.” and “Acts of self-harm are a form of communication about their situation.” (positive attitudes) and disagreed statistically significantly less with the statements “Self-harm may be a form of reassurance for the individual that they are really alive and human.” (positive attitude) and “I feel to blame when my clients self-harm.” (negative attitude) (Table 3).

The most differences were reported with regard to the *type of working environment*. The nurses employed on psychotherapeutic units in comparison to those working in non-psychotherapeutic units agreed statistically significantly more with the statements: “I acknowledge self-harming clients’ qualities.” and “Self-harming individuals can learn new ways of coping.”, both assessing positive attitudes. They disagreed statistically significantly more with the statements: “There is no way of reducing self-harm behaviors.” and “Self-harm is a serious moral wrongdoing.”, both assessing negative attitudes. The nurses employed on psychotherapeutic units disagreed statistically significantly less with the statement “I feel to blame when my clients self-harm.”, assessing negative attitude (Table 3).

## Discussion

The present study aimed to determine the emotions and attitudes of nurses working in different psychiatric settings towards adolescents and young adults with NSSI and their relationship to gender, workplace, education and religious status.

Our results showed that nurses working with individuals who engage in NSSI in the settings of a university psychiatric hospital, experience the least negative of assessed negative emotions and share mostly positive attitudes towards these patients.

They most commonly feel powerless and uncertain, but not angry or despairing and they were undecided about feeling afraid. These findings are in line with the phenomenological studies of interviews with eight community psychiatric nurses reporting feelings of anxiety, frustration, hopelessness, uncertainty and responsibility while working with adults and adolescents who self-injured [32]. Another study on trainee counselors also revealed their struggles with regulating their own emotions when working with clients with NSSI, especially fear, shock and uncertainty, which could all influence their treatment of patients [33]. Our nurses on psychotherapeutic units, however, were not as afraid while working with these patients as those in acute and outpatient settings, which could reflect their stronger theoretical background in the dynamics of NSSI, more experience and knowledge of how to react, as well as the effects of regular supervision as described by the interviewed mental health nurses [32].

With regards to attitudes, the nurses working with young patients with NSSI within our psychiatric hospital reported on average very positive attitudes towards these patients and disagreed with the negative attitudes. However, they also disagreed with the statement “I find it rewarding to care for self-harming clients”. The positive attitudes were expected, since the existing studies reported more positive attitudes in mental health nurses than in professionals from other medical or non-medical settings [15, 34]. The interpretation of not finding care for these patients rewarding is somewhat more intriguing. Many studies report, however, that other professionals as well as nurses working with clients who self-harm often feel incompetent and inadequate [32, 34]. They may find these patients difficult to manage and for these reasons sometimes even avoid them [36]. Specifically, in relation to these emotions, the mental health nurses commonly express the need for external supervision or support from their team [25, 32].

There were no significant differences in experienced emotions with regards to gender. While the female nurses expressed even more positive and less negative attitudes towards the young patients with NSSI than male, they disagreed less about feeling to blame when a patient self-harmed. Our findings are in line with the results of Dickinson et al. who reported more negative attitudes of male than female nurses working in secure environments with patients with NSSI [19]. Other authors report that female mental health nurses felt less effective with these patients [37].

Those with higher education felt more powerless, uncertain and despairing compared to those with lower education, although there were no differences with regards to education in the attitudes the nurses described. These are both contrary to the findings of studies on attitudes, where the attitudes of the staff improve with education or qualification [18, 20–23, 37]. One possible reason could be that the nurses with higher education also felt more responsibility for the improvement of their patients, which in turn could produce reported emotions when working with chronically relapsing patients, similarly to reports by Thompson [32]. The other possible explanation for the discrepant findings in the present study is that the more education in nursing in Slovenia as assessed by the present study (higher education meaning college, university or postgraduate education) does not mean that the nurses are better educated on the nature and modes of treatment of patients with mental disorders or specifically, NSSI. As already reported, only specific tailored education programs enabled the participants to feel more empathetic when treating patients who self-harmed [18, 21].

Indeed, nurses working in psychotherapeutic settings were significantly less likely to experience fear, uncertainty and despair as compared to those working in acute and outpatient psychiatric settings. Their attitudes were also significantly more positive and less negative. This finding could be explained by more experience and theoretical knowledge about the specific pathology, more experience of successful treatments as well as regular opportunities for supervision and intervention by the senior supervisors and members of the psychotherapy team working on psychotherapy wards with the nurses. These experiences may make the nurses less vulnerable to negative emotions, more empathetic and able to connect and experience the patient as a person. On the other hand, the time pressures and more severe crises on the acute wards and less support from the team members in the outpatient settings could make the exposed nurses more vulnerable. A similar observation was reported in studies using specific educational interventions and noted in the professional recommendations [2, 18, 20–22, 25, 32]. Namely, the mental health nurses themselves are the ones most commonly expressing the need for supervision [32], although the trainee counselors under supervision were somewhat disappointed by the experience [33]. Accordingly, our results show a positive trend in nurses’ attitudes and emotions in the psychotherapy settings.

In Slovenia, a former communist country, religiousness used to be actively discouraged. With the former Yugoslav republics’ changes in demographics and changes in the healthcare system, religious support is becoming ever more important in the patient care [38, 39]. There were no significant differences in experienced emotions nor attitudes with regards to whether the nurses were actively religious or not. This is contrary to the findings of Neville and coworkers assessing nurses’ attitudes toward suicide, who reported religion being a significant predictor of positiveness towards medical-surgical inpatients, the most positive being Protestants, followed by Roman Catholic and other Christians [40]. In Slovenia, most of the religiously active population still declare themselves Roman Catholic (57.8% in the last population census), a minority follow other religions (2.3% Greek orthodox, 2.4% Muslim, 0.8% protestant and 0.2% other religions), 3.5% believing but not belonging to a religion, 10.2% atheist and > 15% not willing to declare [41]. The studies are not completely comparable. Our study did not try to determine the emotions and attitudes of nurses of different religions but rather the different levels of active religiousness in mostly Christian nurses, the Neville et al. study assessed attitudes toward attempted suicide and suicide, which could also be the reason why they found differences based on religiousness. In contrast with NSSI, suicidal behavior may conflict more strongly with basic Christian beliefs.

### Limitations of the study

Firstly, the design of our study was cross-sectional, which, in comparison with longitudinal follow-up, can only produce a present state impression of the studied subject. Secondly, our study included only nurses from one university psychiatric hospital, which lowers the sample size and applicability to other hospital or outpatient settings. Thirdly, our results regarding the emotions may be limited, since the respondents were only given the possibility of answering using the five emotions given in the questionnaire. The study could reveal a wider range of emotions if it were interview-based, as was found when registered nurses described shock, disgust and sadness during treatment of patients with NSSI [32]. Finally, the questionnaires used still need to be validated in the Slovene population.

## Conclusions

This is the first study in Slovenia to look into the relationship of healthcare employees towards adolescents and young adult patients with NSSI as well as, to our knowledge, the first to compare psychiatric-psychotherapeutic and non-psychotherapy settings. Our results show that even though the feelings and attitudes of nurses working within a specialized mental health hospital are not negative, there appear to be some important differences between psychiatric settings that need to be taken into account. Therefore, we recommend that the future directives for nurses working with a young population with NSSI in psychotherapeutic as well as in the acute psychiatric settings should be aimed towards offering more supportive environments in terms of specifically tailored education, regular supervision and team support. In order to provide sufficient evidence of the cost–benefit of such interventions, research into the influence of staff attitudes and emotions on these patients’ care should be encouraged nationally and internationally.

## Data Availability

The datasets used and/or analyzed during the current study are available from the corresponding author upon reasonable request.

## Abbreviations

NSSI: Non-suicidal self-injury
NICE: The National Institute for Health and Care Excellence
DSH: Deliberate self-harm
DSM-5: American Diagnostic and Statistical Manual of Mental Disorders—5^th^ Edition
A&E: Accident and emergency
EBQ: Emotional Burden Questionnaire
SHAS: Self-Harm Antipathy Scale
SD: Standard deviation
